# MicroRNA profiling in post-mortem spinal cord of C9ORF72-related ALS patients reveals molecular pathways involved in motor neuron degeneration

**DOI:** 10.3389/fnins.2026.1741065

**Published:** 2026-01-23

**Authors:** Giorgia Farinazzo, Eleonora Giagnorio, Matteo Marcuzzo, Marco Cattaneo, Claudia Malacarne, Paola Cavalcante, Silvia Bonanno, Emanuela Maderna, Viviana Pensato, Cinzia Gellera, Gianluca Marucci, Samanta Mazzetti, Erika Salvi, Giuseppe Lauria, Stefania Marcuzzo

**Affiliations:** 1 Neuroimmunology and Neuromuscular Diseases Unit, Fondazione IRCCS Istituto Neurologico Carlo Besta, Milan, Italy; 2 Department of Electronics, Information and Bioengineering (DEIB), Politecnico di Milano, Milan, Italy; 3 Department of Advanced Diagnostics, Laboratory of Diagnostic and Molecular Research, Fondazione IRCSS Istituto Nazionale dei Tumori, Milan, Italy; 4 ALS Centre, 3rd Neurology Unit, Fondazione IRCCS Istituto Neurologico Carlo Besta, Milan, Italy; 5 PhD Program in Pharmacological Biomolecular Sciences, Experimental and Clinical, University of Milan, Milan, Italy; 6 Neurophatology Unit, Fondazione IRCCS Istituto Neurologico Carlo Besta, Milan, Italy; 7 Unit of Medical Genetics and Neurogenetics, Fondazione IRCCS Istituto Neurologico Carlo Besta, Milan, Italy; 8 Movement Disorders Unit, Fondazione IRCCS Istituto Neurologico Carlo Besta, Milan, Italy; 9 Data Science Center, Fondazione IRCCS Istituto Neurologico Carlo Besta, Milan, Italy; 10 Computational Multi-Omics of Neurological Disorders (MIND) Lab, Fondazione IRCCS Istituto Neurologico Carlo Besta, Milan, Italy; 11 Department of Medical Biotechnology and Translational Medicine, University of Milan, Milan, Italy; 12 Brain-targeted Nanotechnologies (BraiNs) Lab, Fondazione IRCCS Istituto Neurologico Carlo Besta, Milan, Italy

**Keywords:** C9ORF72-amyotrophic lateral sclerosis, human post-mortem spinal cord tissue, microRNAs, motor neuron, target genes

## Abstract

**Introduction:**

Amyotrophic lateral sclerosis (ALS) is a fatal neurodegenerative disorder causing progressive motor neuron death in cortex, brainstem and spinal cord. The most common genetic cause is the G4C2 hexanucleotide repeat expansion in the non-coding region of exon 1 of C9ORF72, accounting for ~40% of familial and ~7% of sporadic ALS. RNA dysregulation is increasingly recognized as a key contributor to ALS pathogenesis. This study aimed to identify specific microRNAs (miRNAs) involved in motor neuron degeneration in C9ORF72-ALS.

**Methods:**

We profiled 754 miRNAs in human post-mortem spinal cord tissue from C9ORF72-ALS patients and healthy donors. Laser capture microdissection isolated ventral horn regions, and in silico target prediction identified potential genes and pathways regulated by differentially expressed miRNAs. Target genes were validated by Real time PCR.

**Results:**

Two subsets of miRNAs were exclusively expressed in ventral horn regions: miR-200b-3p and miR-346 in C9ORF72-ALS patients, and miR-30d-5p, miR-106b-5p and miR-135a-5p in healthy donors. Target prediction and molecular analysis identified putative genes and pathways linked to cell death, inflammation, protein metabolism, DNA modification, excitotoxicity, autophagy and vesicles trafficking.

**Discussion:**

This study identifies specific miRNAs and their target genes as key molecules in motor neuron degeneration in C9ORF72-ALS. Restoring their expression could represent a therapeutic approach for ALS.

## Graphical abstract

**Figure:**
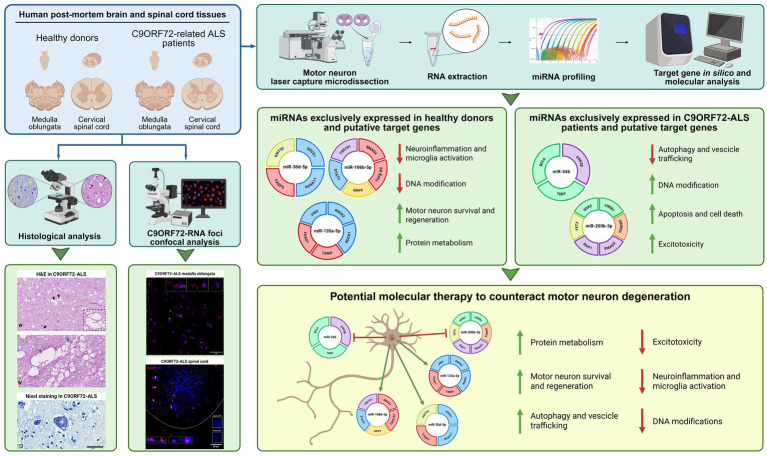

## 1 Introduction

Amyotrophic lateral sclerosis (ALS) is a fatal adult-onset disease characterized by upper and lower motor neuron degeneration occurring in about 85% of patients as sporadic and in 15% as familial with different patterns of inheritance (Feldman et al., 2022). Multiple pathogenic mechanisms have been proposed, including excitotoxicity, aberrant protein aggregation, defective axonal transport, altered RNA metabolism, mitochondrial dysfunction and oxidative stress (Bradford and Rodgers, 2024; Nguyen et al., 2019; Barber and Shaw, 2010). Among more than 40 genes associated with ALS, including superoxide dismutase 1 (SOD1), TAR DNA-binding protein 43 (TARDBP), and FUS RNA-binding protein (FUS; Kirola et al., 2022), the most common genetic cause of the disease is the G4C2 hexanucleotide repeat expansion (HRE) in the non-coding region of exon 1 of the C9ORF72 gene, which accounts for about ~40% of familial ALS and ~7% of sporadic ALS (Raguseo et al., 2023; Ruffo et al., 2025). The pathogenic mechanisms by which HRE in the C9ORF72 gene causes ALS are not fully understood. Three distinct and not mutually exclusive mechanisms have been suggested, including (i) loss-of-function of endogenous C9ORF72 protein that could affect endosomal trafficking and autophagy pathways; (ii) gain-of-function RNA-toxicity caused by an unconventional protein translation mechanism (RAN); (iii) gain-of-function toxicity caused by dipeptide repeat products (DRPs) synthesized by HRE translation (Balendra and Isaacs, 2018). Recently, the key DNA-binding protein death domain-associated protein 6 (DAXX) has been found to recognize C9ORF72 HRE and cause global chromatin remodeling and epigenetic dysregulation in cells from ALS and frontotemporal dementia (FTD) patient (Liu and Rossoll, 2023). While RNA dysregulation has been recognized as a key change for ALS pathogenesis, the role of microRNAs (miRNAs) as molecular modulators of gene expression in motor neurons has been emerging (Hur et al., 2023; Wang et al., 2024). In particular, cell type–specific alterations in miRNA expression profiles contribute to disease phenotypes and may underlie the selective vulnerability of motor neurons. The therapeutic potential of noncoding RNAs, especially miRNAs, is currently being explored as a strategy to complement and enhance existing treatments. In this context, the neuroprotective properties of specific miRNAs may represent a promising avenue for the development of novel therapeutic approaches for motor neuron diseases (De Paola et al., 2019). Emerging therapeutic strategies, including antisense oligonucleotides, gene therapy, and modulators of RNA metabolism, are being actively explored to target the underlying pathogenic mechanisms of ALS, particularly in genetically defined subtypes such as C9ORF72-ALS (Bono et al., 2025; Rofail et al., 2025). These approaches include small molecules modulating RNA metabolism, antisense therapies, and interventions aimed at restoring cellular homeostasis. In addition, experimental therapies targeting protein aggregation, enhancing autophagy, and modulating neuroinflammation are being investigated to slow disease progression and protect motor neurons (Cattaneo et al., 2025). Integrating molecular profiling studies, including miRNA analysis, with these mechanistic insights may offer new avenues for understanding disease progression and identifying potential therapeutic targets. Such advances underscore the critical role of molecular regulators like miRNAs, which may serve both as biomarkers and as potential therapeutic targets in ALS.

Here, we performed the expression analysis of 754 miRNAs in human post-mortem spinal cord tissue of C9ORF72-related ALS patients and healthy donors. Molecular analysis on laser capture microdissection (LCM) samples of cervical spinal ventral horns, followed by in silico target gene prediction and molecular analysis, allowed the identification of distinct miRNAs and their target genes as crucial molecules potentially involved in motor neuron degeneration.

## 2 Materials and methods

### 2.1 Sample collection

We analyzed post-mortem medulla oblongata and cervical spinal cord tissues of two female C9ORF72-related ALS patients and two sex-matched donors deceased for cerebral hemorrhage, stored at the Fondazione IRCCS Istituto Neurologico “Carlo Besta” neuropathology biobank. The time recorded between decease and post-mortem tissue sampling was within 72 h. Tissues were stored in formalin-fixed paraffin-embedded (FFPE) material.

### 2.2 G4C2 hexanucleotide repeat analysis

Genomic DNA was extracted from post-mortem tissues according to a standard phenol-chloroform procedure. The GGGGCC hexanucleotide repeat in C9ORF72 gene was analyzed by a two-step protocol, including a first PCR amplification step using the genotyping primers previously reported (DeJesus-Hernandez et al., 2011). The normal range fragment length analysis was performed on 2% agarose gel. Samples presenting two distinct amplification products in the normal size range are definitively considered negative. Samples resulting in a single amplification product were further analyzed in a second step by the repeat-primed polymerase chain reaction method (AmplideX PCR/CE C9ORF72 Kit (RUO)—Asuragen, Biotechne—Minneapolis—MN, United States) on a 3100XL ABI Prism Genetic Analyzer (Thermo Fisher Scientific—Waltham—MA, United States). The presence of C9ORF72 repeat expansion was assigned when the sample displayed a typical electropherogram profile with decaying stutter amplification peaks. This method is highly sensitive and robust, single-tube, 3-primer C9ORF72 PCR reagents that can flag all expanded samples irrespective of length and provide accurate sizing up to ~145 repeat units using capillary electrophoresis.

### 2.3 Histological analysis of human post-mortem tissues

We obtained 5 μm-thick consecutive sections of medulla oblongata and cervical spinal cord tissues from both ALS and healthy donor post-mortem samples. Sections were then stained using hematoxylin & eosin (H&E) staining protocol for FFPE tissues, applying the following incubation steps: Bioclear (1 min) twice, 100% EtOH (1 min) twice, 95% EtOH (1 min) twice, wash in H2O; hematoxylin (1 min); two washes in H2O; eosin (40 s); two washes in H2O; 70% EtOH (1 min); 90% EtOH (1 min); 100% EtOH (1 min). To perform H&E histological analysis, tissue images were acquired using NanoZoomer-XR (Hamamatsu Photonics K. K.—Hamamatsu City, Japan) digital slide scanner 3.0.

Consecutive sections were counterstained with thionin to further visualize spinal cord cytoarchitecture. The following incubation steps was applyed: Bioclear (1 min) twice, EtOH 100% (1 min) twice, 95% EtOH (1 min) twice, wash in H2O; thionin (5 min); wash in tap H2O; 70% EtOH (1 min); 95% EtOH (to differentiate between white and gray matter); 100% EtOH (1 min); Bioclear (1 min); mounting medium Eukitt® (O. Kindler GmbH). To perform Nissl staining histological analysis, tissue images were acquired using Aperio CS2 (Leica Microsystems GmbH—Wetzlar, Germany).

### 2.4 BaseScope assay for RNA foci detection in human post-mortem medulla oblongata and spinal cord tissue

The presence of C9ORF72-related RNA foci was assessed using the BaseScope v2 Assay for the detection of sense G4C2-repeat expansions in FFPE tissues (ACD, Bio-Techne, Minneapolis, MN, United States). Sections of medulla oblongata and cervical spinal cord were baked in a HybEZ™ II dry oven (ACD, Bio-Techne) for 1 h at 60 °C. Tissue deparaffinization was performed with two incubations in xylene (5 min each, RT), followed by two incubations in 100% EtOH (2 min each, RT), and drying for 10 min at 60 °C. Staining was conducted according to the BaseScope v2 protocol using a target-specific GGGGCC probe and a negative control probe (bacterial dihydrodipicolinate reductase, DapB). Slides were first treated with RNAscope Hydrogen Peroxide (10 min, RT) to block non-specific binding sites, then immersed in RNAscope Target Retrieval Reagent (15 min, 99 °C) to unmask the target sites. Afterwards, they were transferred to 100% EtOH (3 min) and dried completely overnight. The following staining steps were performed using the HybEZ™ Humidity Control Tray (ACD, Bio-Techne) to prevent desiccation between incubations: Protease IV solution (30 min, 40 °C), G4C2 probe hybridization (120 min, 40 °C), Amp1 (30 min, 40 °C), Amp2 (30 min, 40 °C), Amp3 (15 min, 40 °C), Amp4 (30 min, 40 °C), Amp5 (30 min, 40 °C), Amp6 (15 min, 40 °C), Amp7 (30 min, RT), and Amp8 (15 min, RT). Fast RED solution (1:60 ratio of Fast RED-B to Fast RED-A) was then applied for 10 min at RT for signal detection. Nuclei were counterstained with DAPI (1:1000, 10 min, RT). Slides were dried at 60 °C for 15 min and mounted with FluorSave Reagent. Confocal fluorescence images were acquired with a laser-scanning microscope Eclipse TE 2000-E (Nikon Inc.) and analyzed using EZ-C1 3.70 (Nikon Inc.) and Fiji v1.53 (ImageJ, Fiji). C9ORF72-RNA foci quantification was assessed using the Fiji-ImageJ software, according to manufacturer’s protocol. Two images for each tissue type were quantified.

### 2.5 Laser-capture microdissection

Leica LMD7 Laser Microdissection Microscope (Leica Microsystems GmbH—Wetzlar, Germany) was used. For each sample, five 5-μm thick consecutive sections were mounted on the LCM membrane slide, stained with H&E and fixed in RNase-free 75–100% EtOH. Slides were left to completely dry in a completely RNase-free environment under a fume hood for 2 h. The hypoglossal nuclei and spinal cord ventral horns were selected using the Leica Laser Microdissection V8.5 software (Leica Microsystems GmbH), cut and pooled in a RNase-free 0.5 mL Eppendorf and resuspended in lysis buffer (Thermo Fisher Scientific—Waltham—MA, United States) to extract RNA for molecular analysis.

### 2.6 RNA extraction from LCM tissue

Each diffuser isolation cap containing LCM sections was treated with the PureLink FFPE Total RNA Isolation Kit (Thermo Fisher Scientific). To lysate the tissue, melting buffer and proteinase K were added to the tube and incubated at 60 °C for 60–80 min in the Eppendorf ThermoMixer® C (Eppendorf S.r.l.—Hamburg, Germany), with occasional agitation. To isolate tissue total RNA, the following procedures were applied: to bind RNA, binding Buffer (L3) and 100% EtOH were added to the sample; the resulting solution was filtered using the kit Spin Cartidge and centrifuged at 800 x g for 1 min; to wash the Spin Cartridge from unwanted molecule, 3 wash steps with Wash Buffer (W5) followed by 15.000 x g centrifugation for 1 min was performed; to elute the sample, the Spin Cartridge was placed in a clean RNA Recovery Tube and RNase-free water, previously heated at 65 °C, was added to the center of the cartridge. After a 2-min incubation, the solution was eluted through 15.000 x g centrifugation for 1 min; a second elution step was performed using the same water on the same Spin Cartridge to collect the maximum amount of RNA possible. RNA concentration and quality was checked using NanoDrop 2000c Spectophotometer (Thermo Fisher Scientific).

### 2.7 MiRNA profiling and data analysis

Total RNA extracted from the obtained LCM sections was reverse transcribed using Megaplex RT primers Human Pool A and B and MultiScribe Reverse Transcriptase Kit (Thermo Fisher Scientific) following the recommended thermal protocol: 40 cycles consisting of 16 °C for 2 min, 42 °C for 1 min and 50 °C for 1 s; hold stage at 85 °C for 5 min; final hold stage at 4 °C to preserve the sample until further use. cDNA, corresponding to 20 ng of total RNA, was pre-amplified with Preamplification Reaction Mix that was prepared according to manufacturer’s instructions (Thermo Fischer Scientific) and following the recommended thermal protocol: hold at 95 °C for 10 min; hold at 55 °C for 2 min; hold at 72 °C for 2 min; 12 cycles consisting of 95 °C for 15 s and 60 °C for 4 min; hold at 99.9 °C for 10 min; final hold stage at 4 °C. Next, the resulting cDNA was combined with TaqManTM Fast Advanced Master Mix and dispensed into each port of the TaqMan Human MicroRNA array card A and B v2.0, following the manufacturer’s instructions. The arrays were run on the Viia 7 Real-Time PCR System (Thermo Fisher Scientific) following the recommended thermal protocol: hold at 92 °C for 10 min, 40 cycles consisting of 95 °C for 1 s and 60 °C for 20 s, final hold stage at 4 °C. Human array A and B cards contained primers for 754 miRNAs, including 3 positive controls and 1 negative control, with the controls run in technical duplicates. For both samples, a pool of replicates A and B was used. All raw real-time PCR data were imported into the DataConnect cloud platform, and the automatic Crt threshold was applied using the Design and Analysis software (DA2; Thermo Fisher Scientific, Design & Analysis 2 software). Only miRNAs with high-quality amplification (AmpStatus = AMP, Amplification Score > 1, and Cq Confidence > 0.8) were included in the downstream analysis. miRNAs not meeting these quality thresholds were classified as null and consequently considered as not expressed. To find the most stable miRNAs, in both cards separately we used the user-friendly web-based tool RefFinder^1^ developed for evaluating and screening reference genes/miRNAs from extensive experimental datasets. Expression stability ranking identified hsa-miR-34a-000426 as the most suitable miRNA reference for Pool A and hsa-miR-30e-3p-000422 for Pool B, respectively.

Due to the low number of biological replicates, our analysis relied solely on a qualitative presence/absence assessment of miRNAs, without performing group-wise differential expression testing. miRNAs were considered exclusively expressed if detected in only one condition considering the expression in both replicates of either healthy donors or C9ORF72-ALS patients.

### 2.8 In silico target gene prediction and gene ontology enrichment analysis

A comprehensive in silico pipeline was implemented to identify putative miRNA target genes and characterize their functional relevance. Target prediction was performed by integrating three miRNA–mRNA interaction databases: miRTarBase, TarBase v9 and TargetScan 8.0. For each miRNA, all predicted or experimentally validated interactions were retrieved; only genes predicted by at least two out of the three databases were retained to increase confidence in the interactions.

These genes were further filtered to include only those previously implicated in ALS pathogenesis, based on manual curation of the literature. To annotate candidate miRNAs and their putative target mRNAs, we performed a systematic PubMed search. We included studies reporting experimentally validated miRNA–target interactions, functional assays, or high-confidence bioinformatic predictions supported by experimental evidence, and excluded studies lacking primary data, purely computational predictions, or not relevant to human neurodegenerative disease. For each miRNA–mRNA pair, we recorded the type of supporting evidence, including assay type, experimental model, and outcomes. In cases of conflicting results, both were noted, giving preference to studies with direct experimental validation in human samples or well-established models. In addition, genes involved in molecular pathways associated with C9ORF72-related mechanisms (autophagy, nucleocytoplasmic transport, excitotoxicity, RNA metabolism) were prioritized. Functional enrichment analysis was conducted using ClueGO (Cytoscape v3.10.3). Gene Ontology (GO) terms (Biological Process, Molecular Function and Cellular Component) and pathway databases (KEGG 2024 and Reactome 2024) were queried in order to elucidate terms associated with the selected miRNAs.

### 2.9 mRNA quantitative real-time PCR

Total RNA extracted from the LCM captured ventral horn of cervical spinal cord tissue sections derived from the two C9ORF72-ALS patients and two healthy donors, previously examined for miRNA expression, was reverse transcribed using SuperScript Vilo cDNA Synthesis kit (Invitrogen, Thermo Fisher Scientific) and the following thermal protocol: hold stage at 25 °C for 10 min, hold stage at 42 °C for 60 min, hold stage at 85 °C for 5 min, final hold stage at 4 °C. cDNA (corresponding to 10 ng total RNA) was amplified by quantitative real-time PCR, in duplicate, using TaqMan Fast Advanced Master Mix and TaqMan gene expression assays specific for CASP3, KMT2C, HOXA11, SOCS1, STAT3, TGFBR2, SMAD4, TSG101, DPP9, JAK2, ROCK1, ROCK2, TXNIP, FOXO1, SOX2, CREB1, GRIN2A, SMAD2, BAP1, TET3, BCL6, GSK3B, TERT genes on ViiA7 Real-time PCR system (Thermo Fisher Scientific). 18S has been chosen as housekeeping (Thellin et al., 1999). Specific TaqMan assay IDs are reported in Supplementary Table 1. The following thermal protocol was used: hold stage at 95 °C for 20 s, 40 cycles consisting of 95 °C for 1 s and 60 °C for 20 s, final hold stage at 4 °C. mRNA expression levels were normalized against 18S, and relative expression was calculated using the 2^−∆Ct^ method. The Heatmapper tool (https://www.heatmapper2.ca/) was used to perform hierarchical clustering of the columns following standard procedures for expression heatmap visualization (Babicki et al., 2016; Kernick et al., 2025). The Expression module was employed, with the Euclidean distance metric to measure pairwise similarity and the Average Linkage method to define cluster relationships. This approach follows standard procedures for visualizing relative expression patterns while maintaining reproducibility.

## 3 Results

### 3.1 Pathophysiological changes in C9ORF72-ALS patient’s post-mortem medulla oblongata and spinal cord tissues

The G4C2 hexanucleotide repeat analysis showed the presence of G4C2-rich repeat expansion in ALS tissues (Supplementary Figure 1).

**Figure 1:**
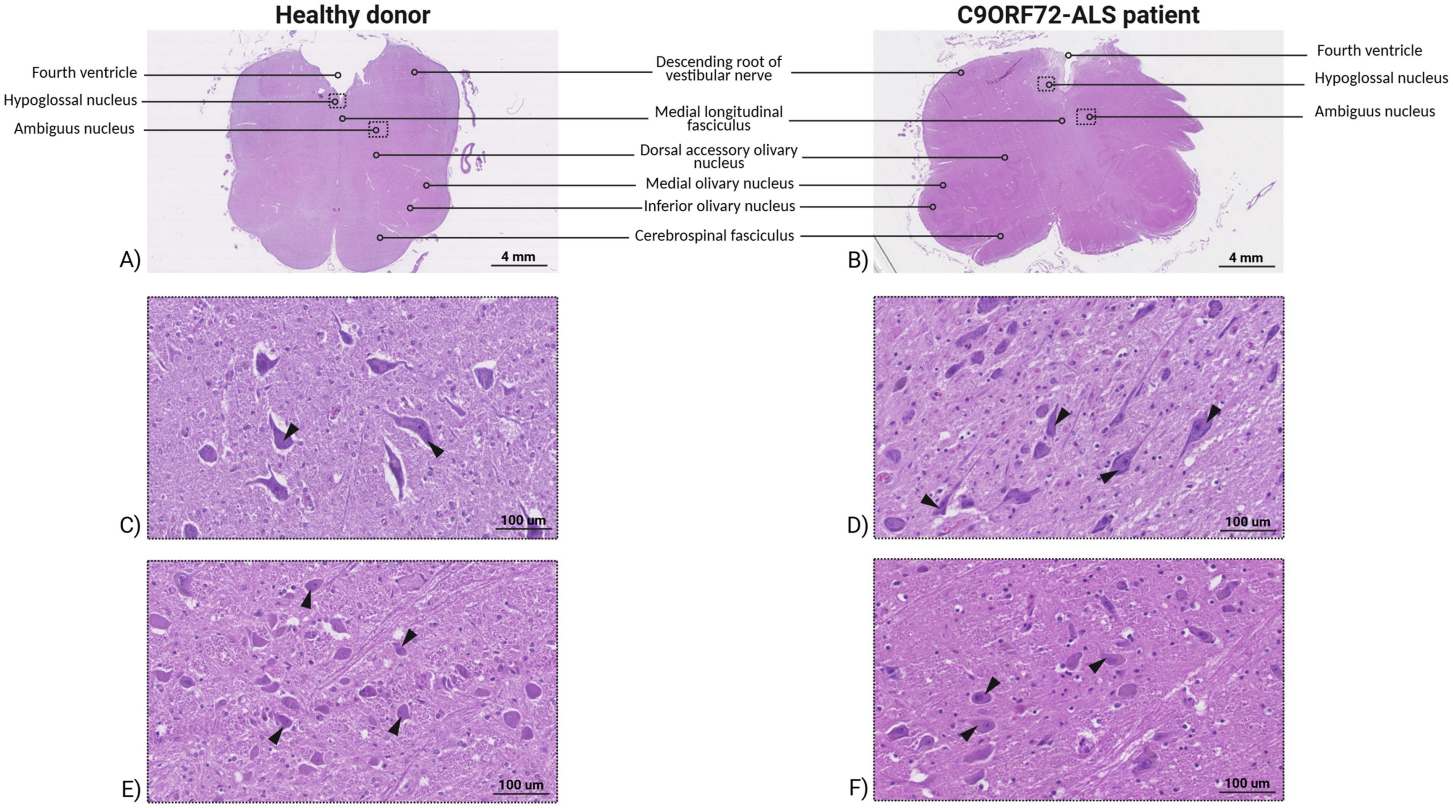
Section of human post-mortem medulla oblongata tissue of healthy donor and C9ORF72-related ALS patient stained by H&E. Human post-mortem medulla oblongata of healthy donor **(A)** and C9ORF72-related ALS patient **(B)**. Magnification 10x, scale bar 4 mm. Hypoglossal **(C)** and ambiguous **(D)** nucleus of healthy donor, black arrowheads indicate motor neurons. Magnification 300x, scale bar 100 μm. Hypoglossal **(E)** and ambiguous **(F)** nucleus of C9ORF72-related ALS patient, black arrowheads indicate motor neurons. Magnification 300x, scale bar 100 μm.

We focused on the hypoglossal and ambiguous nuclei of post-mortem medulla oblongata tissue sections, which can be detected close to the fourth ventricle (Figure 1). At both lower and higher magnification, no pathological changes were identified in C9ORF72-ALS samples, consistent with the spinal onset and progression of the disease at the time of the death. To confirm this, we quantified the amount of stained motor neurons in both C9ORF72-ALS patients’ and healthy donors’ medulla oblongata motor nuclei. Healthy donor’s hypoglossal and ambiguous nuclei showed, respectively, an average of 76.19 and 104.04 motor neurons, while the same regions in C9ORF72-ALS patient’s medulla oblongata displayed an average of 83.09 and 99.25 stained motor neurons, respectively.

**Figure 2:**
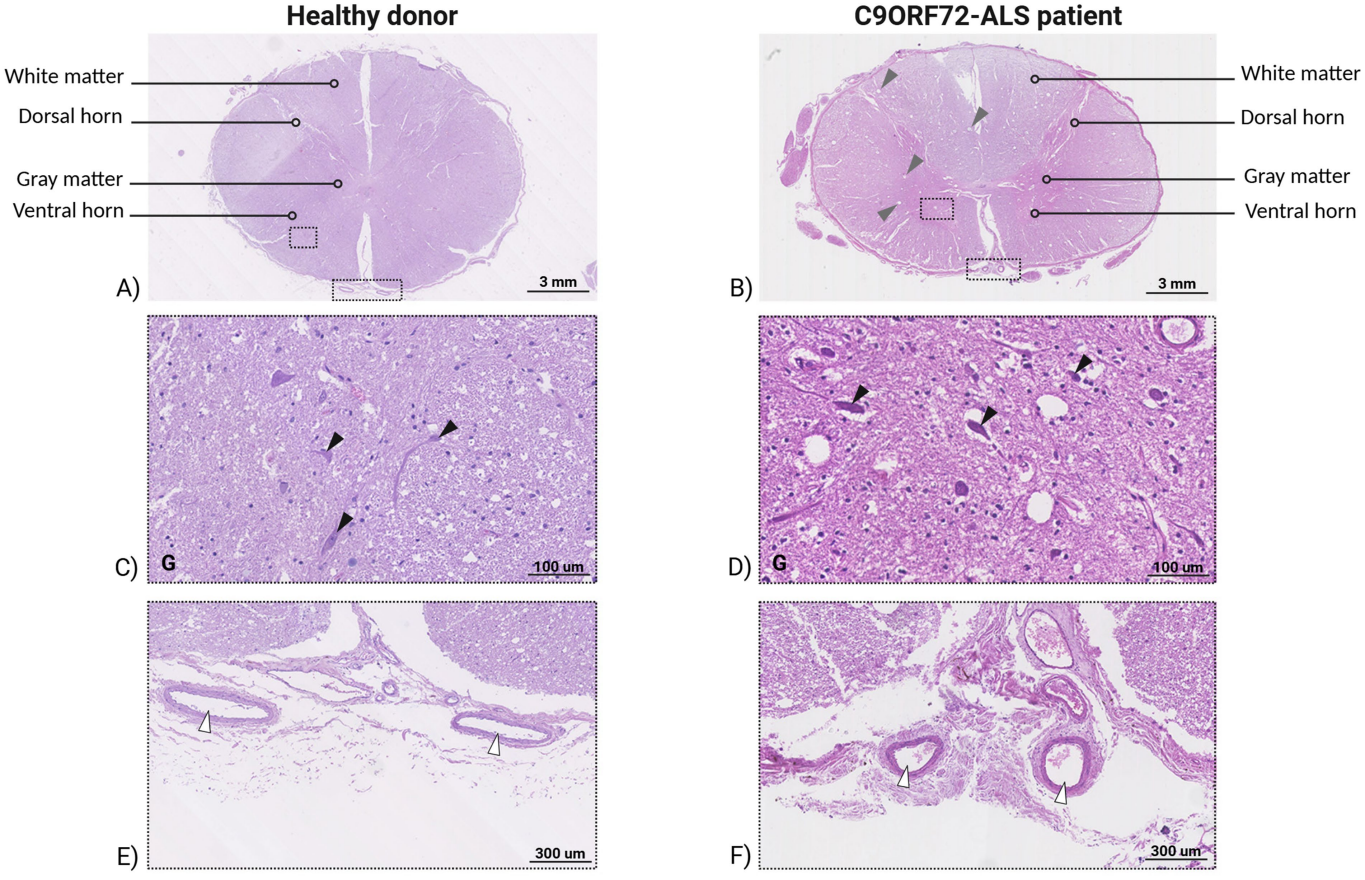
Sections of human post-mortem ventral spinal cord tissue of healthy donor and C9ORF72-related ALS patient stained by H&E. Human post-mortem spinal cord tissues of healthy donor **(A)** and C9ORF72-related ALS patient **(B)**. Magnification 10×, scale bar 3 mm. Representative image of gray matter of ventral spinal cord of healthy donor **(C)** and C9ORF72-related ALS patient **(D)**. Black arrowheads indicate motor neurons. Magnification 300×, scale bar 100 μm. Anterior vein and artery in healthy donor **(E)** and C9ORF72-related patient **(F)** around the ventral spinal cord region. White arrowheads indicate blood vessels. Magnification 100×, scale bar 300 μm.

**Figure 3:**
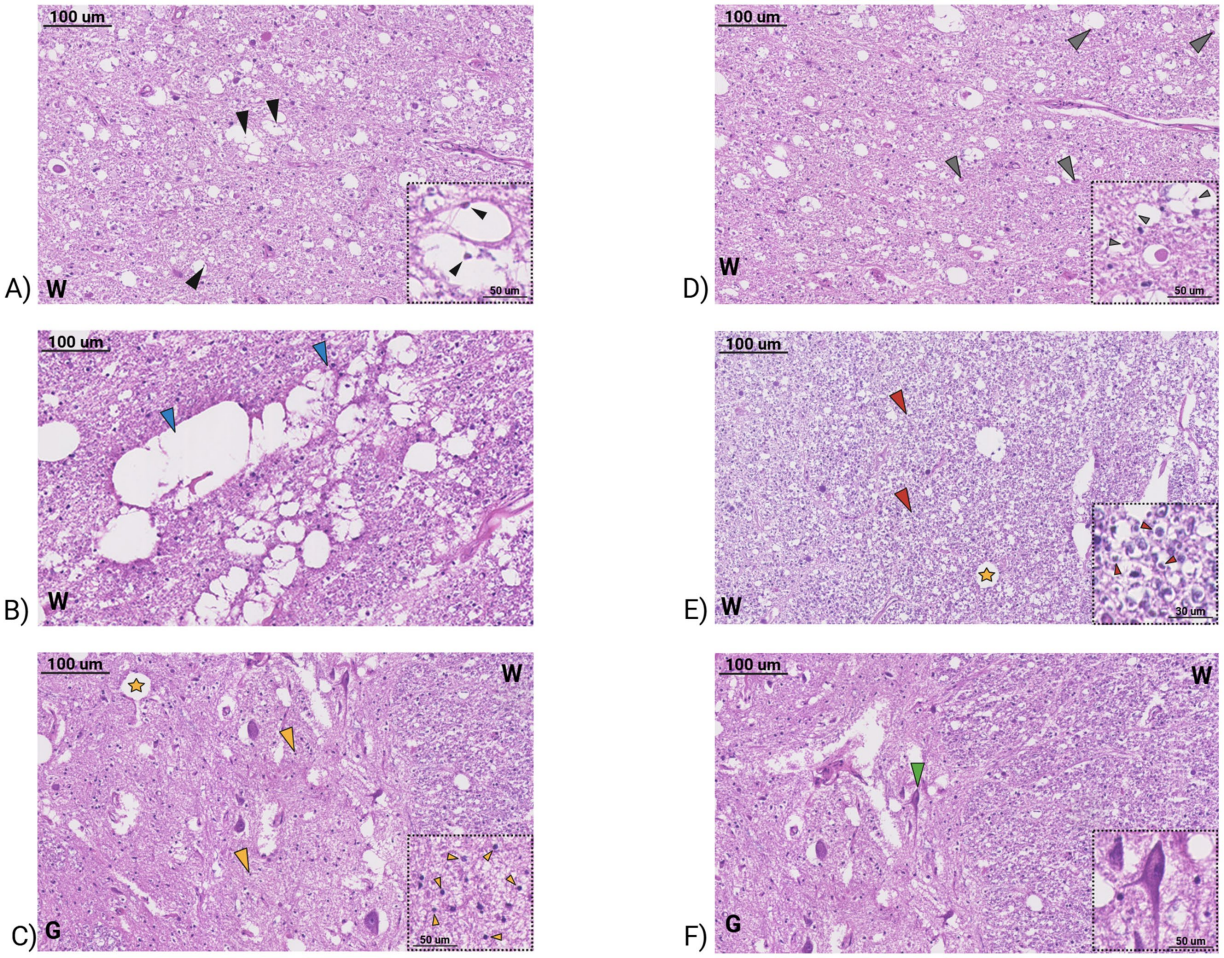
Section of human post-mortem ventral spinal cord tissue of C9ORF72-related ALS patient stained by H&E. **(A)** White matter (W) of ventral spinal cord, black arrowheads indicate axon degeneration. Magnification 150x, scale bar 100 μm. The inset shows enlargement of degenerating axons (black small arrowheads). Magnification 450x, scale bar 50 μm. **(B)** White matter (W) of ventral spinal cord, light blue arrowheads indicate coalescing vacuole area. Magnification 150x, scale bar 100 μm. **(C)** Gray (G) and white (W) matter of ventral spinal cord, yellow arrow heads indicate activated glial cells and yellow star indicates large cavitation area. Magnification 150x, scale bar 100 μm. The inset shows enlargement of active glial cells in gray matter (yellow small arrowheads). Magnification 450x, scale bar 50 μm. **(D)** White matter (W) of ventral spinal cord, gray arrowheads indicate axon swelling. Magnification 150x, scale bar 100 μm. The inset shows enlargement of swelling axons (gray small arrowheads). **(E)** The panel shows enlargement of active glial cells in white matter (red small arrowheads) and yellow star indicates large cavitation area. Magnification 450x, scale bar 50 μm. **(F)** Gray matter (G) and white matter (W) of ventral spinal cord, green arrowheads indicate pyknotic nuclei of degenerating motor neurons. Magnification 150x, scale bar 100 μm. The inset shows enlargement of motor neuron pyknotic nuclei (green small arrowheads). Magnification 450x, scale bar 50 μm.

In the same two patients, the ventral horn regions of the cervical spinal cord showed an altered architecture with disassembled motor neurons, numerous vacuoles both in the gray and white matter at low magnification (Figure 2). At higher magnification, motor neuron bodies showed reduced size and altered morphology consequently leaving empty spaces, pyknotic nuclei indicating chromatin and nucleus shrinkage during apoptosis and necrosis processes, while the whole sections tissues showed sponge-like degenerating features. Coalescing vacuole areas and big cavitations were found in both white and gray matter, indicating the complete degeneration of motor neurons in the ventral spinal cord. In addition, abundant activated glial cells were observed both in gray and white matter of ventral spinal cord sections (Figure 3).

**Figure 4:**
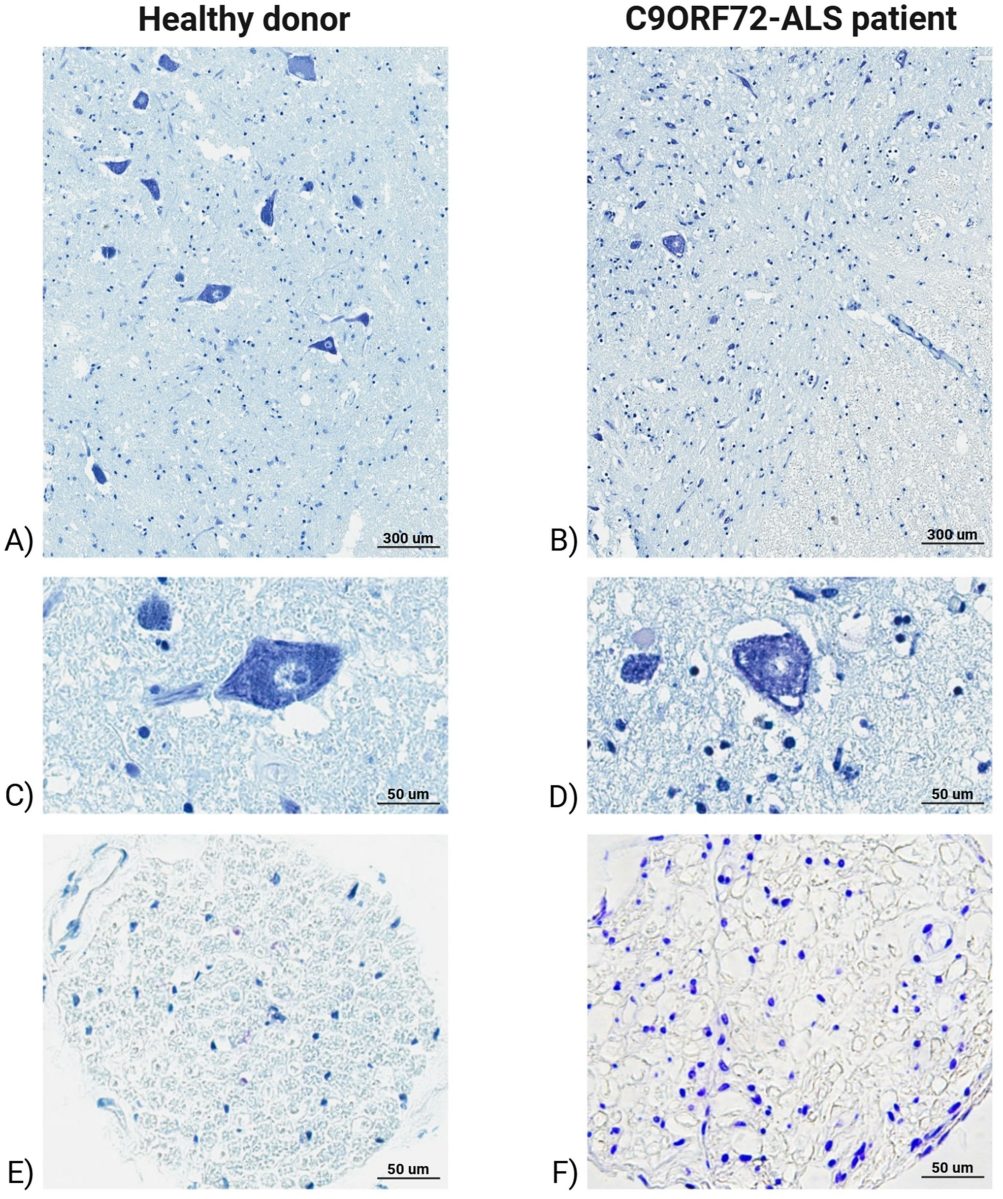
Sections of human post-mortem ventral spinal cord and peripheral nerve tissues of healthy donor and C9ORF72-related ALS patient stained with Nissl. Ventral spinal cord tissues of healthy donor **(A)** and C9ORF72-related ALS patient **(B)**. Magnification 20x, scale bar 300 μm. **(C)** The image shows motor neurons in the ventral spinal cord of healthy donors with the classical aspect of the negative nucleus and intensely positive nucleolus and tigroid substance of the motoneurons. Magnification 40x, scale bar 50 μm. **(D)** The image indicates the small motor neurons in the ventral spinal cord of C9ORF72-related ALS patient showing different size circumferential cytoplasmic inclusions. Magnification 40x, scale bar 50 μm. **(E)** Peripheral nerve tissues of healthy donor with numerous fibers with a similar caliber. Magnification 20x, scale bar 50 μm. **(F)** Peripheral nerve tissues of C9ORF72-related ALS patient showing an altered band irregular morphology, associated to a great amount of glial cells nuclei. Magnification 20x, scale bar 50 μm.

The Nissl staining displayed small motor neurons with different circumferential cytoplasmic size and “skein-like” inclusions in the ventral spinal cord tissues of C9ORF72-related ALS patient (Figure 4). In addition, the ALS peripheral nerve sections showed an altered band irregular morphology, associated to a great amount of glial cells nuclei (Figure 4). Neuronal loss in C9ORF72-ALS patients compared to healthy donors was additionally assessed by stained motor neuron quantification, which resulted in an average of 59.33 motor neurons detected in the ventral horns of cervical spinal cord of ALS-related patients, whereas healthy donors showed the presence on average of 81.17 motor neurons in the same regions.

### 3.2 RNA foci in medulla oblongata and ventral horn of spinal cord tissue of C9ORF72-ALS patients

**Figure 5:**
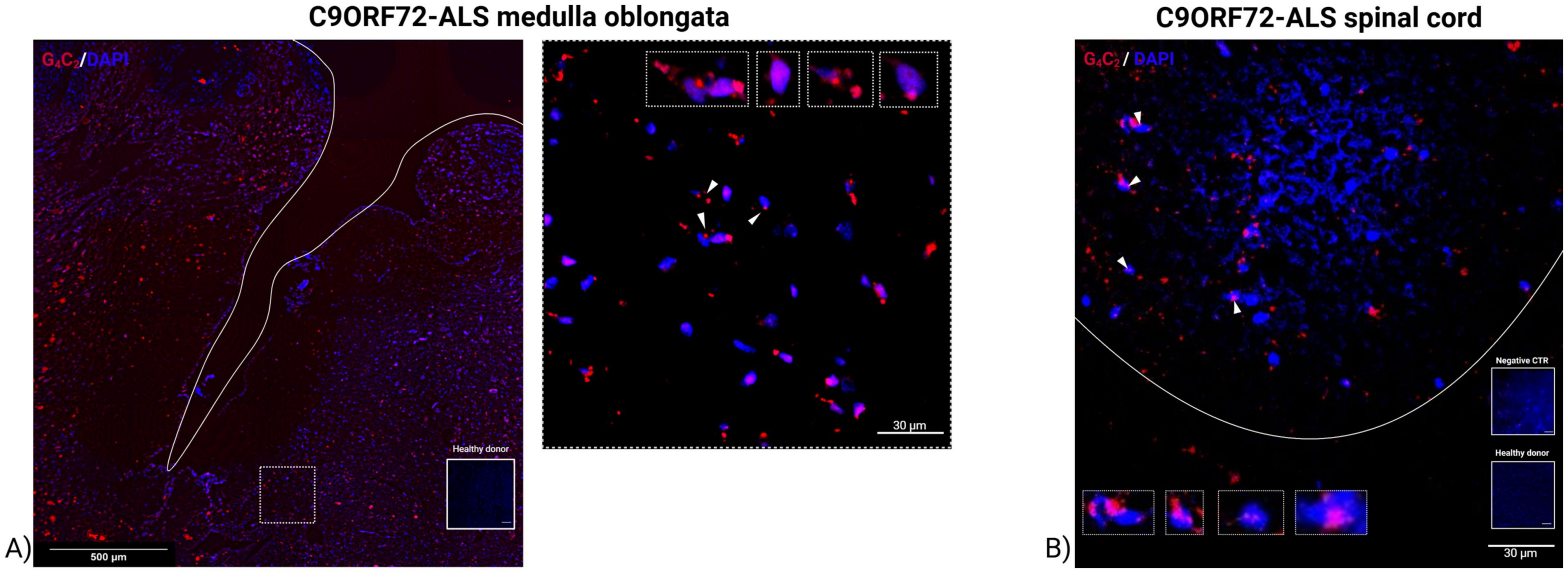
Confocal microscopy images of RNA foci in human post-mortem medulla oblongata and ventral spinal cord tissue of C9ORF72-related ALS patient. Representative images of medulla oblongata **(A)** and ventral horn of the spinal cord **(B)** of C9ORF72-related ALS patient stained for G4C2-rich repeat expansion (red) and DAPI motor neuron nuclei (blue). **(A)** The white line delimits the fourth ventricle area in the medulla oblongata section, while the dashed area delimits the hypoglossal nucleus. Magnification 20x, scale bar 500 μm. White arrowheads indicate the co-localization of G4C2-rich repeat expansion with DAPI-stained nuclei. Magnification 63x, scale bar 30 μm. The insets show the co-localization of G4C2-rich repeat expansion with DAPI-stained bulbar motor neurons. Magnification 150x. **(B)** White arrowheads indicate the co-localization of G4C2-rich repeat expansion with DAPI-stained nuclei. Magnification 63x, scale bar 30 μm. The white line indicates the boundary between white (W) and gray matter (G) of the ventral spinal cord tissue. The insets show the co-localization of G4C2-rich repeat expansion with DAPI-stained nuclei motor neuron. Magnification 150x.

To further investigate the pathological features linked to C9ORF72-ALS pathology, we performed BaseScope Assay RNA scope assay in human post-mortem sections of medulla oblongata and ventral horn of cervical spinal cord of C9ORF72-ALS patients. Motor neurons were stained with a G4C2 red fluorescent probe to detect the presence of RNA foci. As shown in Figure 5, C9ORF72-ALS medulla oblongata and spinal motor neurons displayed aggregates of G4C2 repeats in the nuclei confirming the toxic gain of function of the expiation in the C9ORF72 gene. C9ORF72-RNA foci quantification in motor neurons of C9ORF72-ALS patients revealed an average of 1.9 foci per cell in the ventral horns of cervical spinal cord samples, while motor nuclei in the medulla oblongata displayed 1.5 foci per cell. Although both regions displayed similar RNA foci accumulation, overt motor neuron degeneration was mainly observed in the spinal cord, suggesting a region-specific susceptibility likely due to higher vulnerability of spinal motor neurons to repeat RNA toxicity and impaired RNA metabolism, whereas medullary neurons may activate compensatory mechanisms that delay cell death.

### 3.3 MiRNA expression in healthy donor and C9ORF72-related ALS post-mortem cervical ventral horn tissue

To identify miRNAs potentially implicated in motor neurodegeneration, we performed a miRNA expression profiling of 754 human miRNAs in LCM captured ventral horn of cervical spinal cord tissue sections (Supplementary Figure 2) derived from the two C9ORF72-ALS patients and two healthy donors. Our molecular and bioinformatics analyses revealed a specific group of miRNAs, including miR-10b, miR-30d-5p, miR-93-3p, miR-106b-5p, miR-127-3p, miR-135a-5p and miR-590-5p, exclusively expressed in the ventral horn of cervical spinal cord regions of healthy donors but not in C9ORF72 ALS patients (Table 1). Through an integrated in silico analysis of predicted target genes and a thorough review of the literature (Brett et al., 2011; Gupta et al., 2012; Li et al., 2021; Liu et al., 2016; Martínez-Fábregas et al., 2018; Pang and Hu, 2023; Petrocca et al., 2008; Shu et al., 2022; van Battum et al., 2018; Wang et al., 2020; Xia et al., 2019; Yang et al., 2013). We selected miR-30d-5p, miR-106b-5p and miR-135-5p for downstream analyses, due to their known cellular functions related to autophagy, neuroprotection and axonal regrowth (Table 2; Supplementary Tables 2, 3).

**Table 1:** Summary of miRNAs exclusively expressed in ventral horn of spinal cord tissue sections of healthy donors.

miRNA ID assay	miRNA	Pool	Healthy donors (ΔCt)	C9ORF72-ALS patients (ΔCt)
hsa-miR-10b#-002315	miR-10b	A	2,532	Undetected
hsa-miR-30d-000420	miR-30d-5p	B	2,092	Undetected
hsa-miR-93#-002139	miR-93-3p	B	1,3,335	Undetected
hsa-miR-106b-000442	miR-106b-5p	A	3,0465	Undetected
hsa-miR-127-000452	miR-127-3p	A	2,7,985	Undetected
hsa-miR-135a-000460	miR-135a-5p	A	5,0875	Undetected
hsa-miR-590-001984	miR-590-5p	A	4,2,875	Undetected

List of miRNAs exclusively detected in healthy control samples (*N* = 2). ΔCt values are obtained via the normalization with hsa-miR-34a-000426 values for Pool A and hsa-miR-30e-3p-000422 values for Pool B.

**Table 2:** Summary of the selected miRNAs and their functions elucidated by literature, exclusively expressed in ventral spinal cord tissue sections of healthy donors.

miRNA	Molecular mechanism	Reference
miR-30d-5p	Inhibition of autophagic cell death by targeting BECN1/ATG5 pathway.	Yang et al. (2013) and Shu et al. (2022)
	Inhibition of microglia reactivity and pro-inflammatory factors secretion.	Li et al. (2021)
miR-106b-5p	Neuron survival via activation of the TGFB pathway.	Gupta et al. (2012) and Petrocca et al. (2008)
	Maintenance of adult NSC pool via insulin/IGF signaling.	Brett et al. (2011) and Xia et al. (2019)
	Neuroprotection via inhibition of JAK/STAT pathway.	Martínez-Fábregas et al. (2018) and Pang and Hu (2023)
miR-135a-5p	Stimulation of axonal regrowth.	van Battum et al. (2018) and Wang et al. (2020)
	Neuroprotection and apoptosis inhibition.	Wang et al. (2020) and Liu et al. (2016)

Conversely, we found that miR-200b-3p, miR-346, and miR-1225 were exclusively expressed in the ventral spinal cord horns of C9ORF72-ALS patients (Table 3). Subsequently, only two miRNAs were selected according to predicted target genes and evidence reported in the literature (Fletcher et al., 2022; Fu et al., 2019; Guo et al., 2020; Jiang et al., 2023; Kmetzsch et al., 2021; Trümbach and Prakash, 2015; Wei et al., 2015), since the increased expression of miR-200b-3p and miR-346 could be associated with the modulation of autophagy machinery, ER stress, cell survival, excitotoxicity, DNA modifications and vesicle trafficking (Table 4; Supplementary Tables 2, 3).

**Table 3:** Summary of miRNAs exclusively expressed in ventral horn of spinal cord tissue sections of C9ORF72-ALS patients.

miRNA ID assay	miRNA	Pool	Healthy donors (ΔCt)	C9ORF72-ALS patients (ΔCt)
hsa-miR-200b-002251	miR-200b-3p	A	Undetected	2.1225
hsa-miR-346-000553	miR-346	A	Undetected	−0.3195
hsa-miR-1225-3P-002766	miR-1225	B	Undetected	−2.4735

List of miRNAs exclusively detected in C9ORF72-ALS samples (*N* = 2). ΔCt values were obtained via the normalization with hsa-miR-34a-000426 values for Pool A and hsa-miR-30e-3p-000422 values for Pool B.

**Table 4:** Summary of the selected miRNAs and their functions elucidated by literature, exclusively expressed in ventral spinal cord tissues sections of C9ORF72-ALS.

miRNA	Molecular mechanisms	Reference
miR-200b-3p	Modulation of survival-related transcription factors.	Fu et al. (2019) and Kmetzsch et al. (2021)
	Autophagy dysregulation and ER stress induction.	Trümbach and Prakash (2015) and Wei et al. (2015)
miR-346	Regulation of pro-survival genes.	Fletcher et al. (2022), Guo et al. (2020), and Jiang et al. (2023)

### 3.4 Expression patterns of putative miRNA target genes in healthy donor and C9ORF72-related ALS post-mortem cervical ventral horn tissue

**Figure 6:**
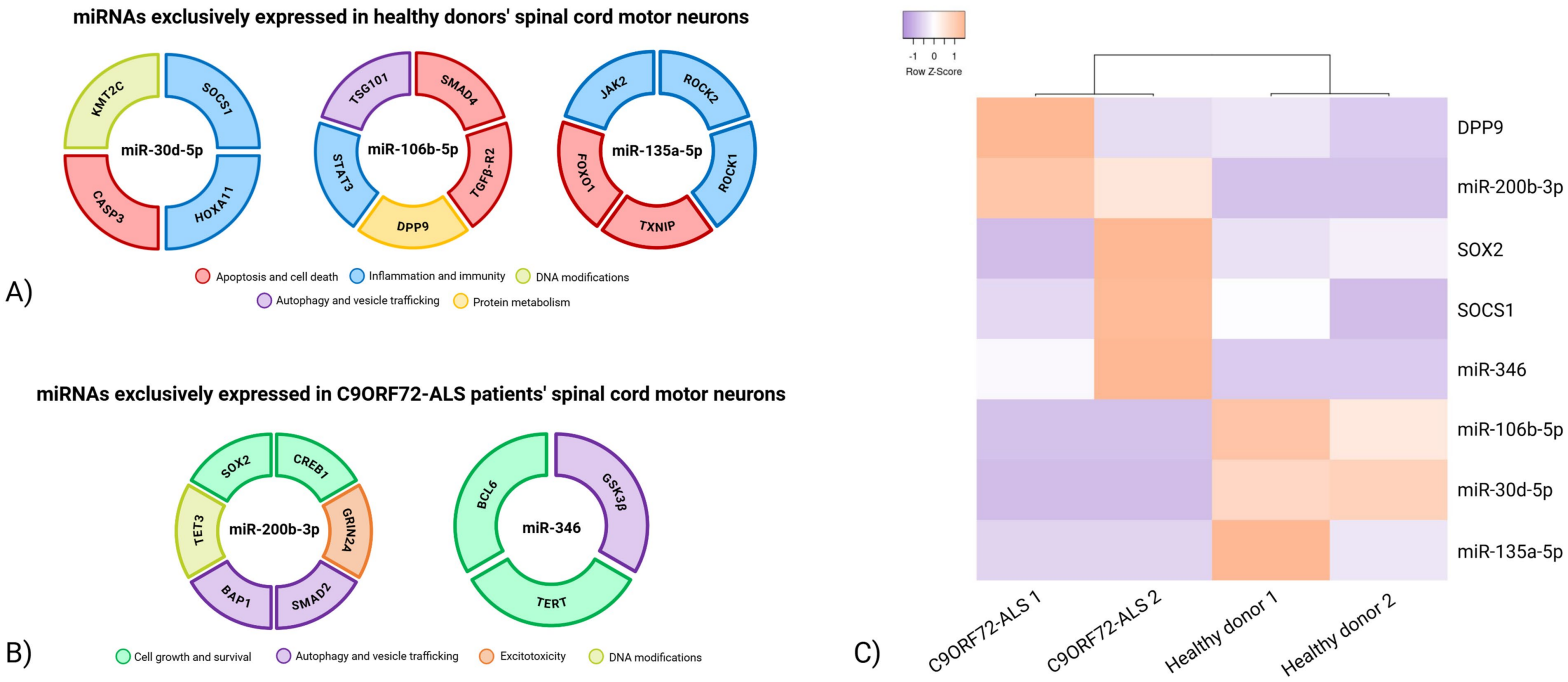
Functional distribution and expression clustering of miRNA target genes in healthy and C9ORF72-ALS spinal cord tissue. **(A)** Putative target genes identified in relation to miRNAs exclusively expressed in spinal cord tissue of healthy donor. Cake graphs indicate the selected miRNAs and target genes with their cellular functions: apoptosis and cell death (red), inflammation and immunity (blue), DNA modifications (light green), autophagy and vesicle trafficking (purple), protein metabolism (yellow). **(B)** Putative target genes identified in relation to miRNAs exclusively expressed in ventral spinal cord tissue of C9ORF72-related ALS patients. Cake graphs indicate the selected miRNAs and target genes with their cellular functions: cell growth and survival (green), excitotoxicity (orange), DNA modifications (light green), autophagy and vesicle trafficking (purple). **(C)** Heatmap of miRNAs and predicted gene targets in healthy donor and C9ORF72-related ALS ventral spinal cord tissue. Expression data are normalized against hsa-miR-34a-000426 for miR-106b-5p and miR-135a-5p, miR-200b-3p and miR-346, with hsa-miR-30e-3p-000422 for miR-30d-5p and with 18S for target genes and expressed as 2^−∆Ct^ values. Light purple indicates down-regulation and orange indicates up-regulation of miRNAs and predicted gene targets, respectively. Hierarchical clustering analysis identifies two clusters of miRNAs and their predicted targets: one characterizing healthy donor spinal cord tissues; the other characterizing C9ORF72-related ALS spinal cord tissue.

Based on miRNA-target prediction databases, Gene Ontology enrichment analysis and previously published studies (Figure 6A; Table 5; Supplementary Table 2), the following target mRNAs and their biological pathways were identified for miRNAs expressed in controls but not C9ORF72-ALS spinal cord: (i) Caspase 3 (CASP3) implicated in apoptosis and cell death pathways (Dafinca et al., 2016); Home box A11 (HOXA11), and Suppressor of cytokine signaling 1 (SOCS1) implicated in inflammation and immunity (Li et al., 2022; Spiller et al., 2018); Lysine Methyltransferase 2C (KMT2C) implicated in DNA modifications (Park et al., 2022), for miR-30d-5p; (ii) Transforming growth factor beta receptor 2 (TGFB-R2), SMAD family member 4 (SMAD4) implicated in apoptosis and cell death pathways (Petrocca et al., 2008; Meroni et al., 2019; Jiang et al., 2005); Tumor susceptibility 101 (TSG101) implicated in autophagy and vesicle trafficking (Kaul et al., 2020; Walker et al., 2016), Signal transducer and activator of transcription 3 (STAT3) implicated in inflammation and immunity (Pang and Hu, 2023; Toral-Rios et al., 2020); Dipeptidyl peptidase 9 (DPP9) implicated in protein metabolism (Finger et al., 2020), for miR-106b-5p; (iii) Forkhead Box O1 (FOXO1) and Thioredoxin interacting protein (TXNIP) implicated in apoptosis and cell death pathways (Tang et al., 2014; Andrés-Benito et al., 2017), Janus kinase 2 (JAK2), Rho associated coiled-coil containing protein kinase (ROCK1 and ROCK2) implicated in inflammation and immunity (Satriotomo et al., 2006; Wang et al., 2020; Tönges et al., 2014), for miR-135a-5p.

**Table 5:** Summary of the selected miRNAs, target genes and their principal molecular mechanisms.

miRNAs	Target gene	Molecular mechanism	Reference
miR-30d-5p	HOXA11	Inflammation and microglia activation via Wnt pathway	Li et al. (2022)
	SOCS1	Inflammation and microglia activation via JAK/STAT pathway	Spiller et al. (2018)
	KMT2C	Histone modification	Park et al. (2022)
	CASP3	Apoptosis	Dafinca et al. (2016)
miR-106b-5p	DPP9	Protein metabolism	Finger et al. (2020)
	STAT3	Inflammation and microglia activation via JAK/STAT pathway	Pang and Hu (2023) and Toral-Rios et al. (2020)
	TGFBR2	TGFB pathway activation	Petrocca et al. (2008) and Meroni et al. (2019)
	SMAD4	TGFB pathway activation	Jiang et al. (2005)
	TSG101	Vesicle trafficking	Kaul et al. (2020) and Walker et al. (2016)
miR-135a-5p	JAK2	Inflammation and microglia activation via JAK/STAT pathway	Satriotomo et al. (2006)
	ROCK1/2	Inflammation and microglia activation via RhoA/ROCK pathway	Wang et al. (2020) and Tönges et al. (2014)
	TXNIP	Apoptosis induction	Andrés-Benito et al. (2017)
	FOXO1	Apoptosis induction	Tang et al. (2014)
miR-200b-3p	SOX2	Pro-survival gene expression	Pandey et al. (2015)
	CREB1	Pro-survival gene expression	Peng et al. (2013)
	TET3	Pro-survival gene expression via DNA demethylation	Yang et al. (2020)
	GRIN2A	Neuron excitability	Gunasekaran and Omkumar (2022)
	BAP1	Protein deubiquitination	Kim et al. (2022)
	SMAD2	Autophagy and protein clearance	Zhou et al. (2020)
miR-346	BCL6	Cell proliferation and survival via Wnt pathway inhibition	Jiang et al. (2023)
	TERT	Neuron protection and survival via	Song et al. (2015)
	GSK3B	Autophagy activation	Guo et al. (2020)

Similarly, integrating target-prediction resources, functional enrichment results and evidence from the literature (Figure 6B; Table 5; Supplementary Table 2), the following target mRNAs and their biological pathways were identified for miRNAs exclusively expressed in C9ORF72-ALS spinal cord tissue: (i) CAMP responsive element binding protein 1 (CREB1), SRY-Box Transcription Factor 2 (SOX2) implicated in cell growth and survival (Peng et al., 2013; Pandey et al., 2015); Glutamate ionotropic receptor NMDA type subunit 2A (GRIN2A) implicated in excitotoxicity (Gunasekaran and Omkumar, 2022); BRCA1 associated protein 1 (BAP1), SMAD2 implicated in autophagy and vesicle trafficking (Kim et al., 2022; Zhou et al., 2020); Tet methylcytosine dioxygenase 3 (TET3) implicated in DNA modifications (Yang et al., 2020), for miR-200b-3p; (ii) BCL6 Transcription Repressor (BCL6), Telomerase reverse transcriptase (TERT) implicated in cell growth and survival (Jiang et al., 2023; Song et al., 2015); Glycogen synthase kinase 3 beta (GSK3β) implicated in autophagy and vesicle trafficking (Guo et al., 2020) for miR-346.

Gene expression levels were quantified in LCM ventral horn spinal cord sections of the same healthy donors and C9ORF72-related ALS patients. Molecular analysis by RT-PCR revealed a trend toward lower expression of SOCS1 and DPP9 genes in healthy tissues. Although preliminary, these results suggest that the inflammatory signaling pathway leading to SOCS1 activation, as well as the protein metabolism regulated by DPP9, is not engaged in control tissues, unlike in C9ORF72-related ALS, where both pathways appear to be upregulated (Figure 6C). Moreover, SOX2 expression was elevated in C9ORF72-related ALS tissues compared to controls, suggesting a pro-survival response aimed at counteracting motor neuron degeneration (Figure 6C). Hierarchical clustering was performed on the samples, as visualized by the column dendrograms of the heatmap and identified two clusters of miRNAs and their predicted targets: one characterizing healthy donor tissue; the other characterizing C9ORF72-related tissues. This separation reflects the expected divergence between C9ORF72-ALS and healthy donor tissues, given that only those genes that were actually expressed were included and that the selected miRNAs and their predicted targets display condition-specific expression patterns. Therefore, the heatmap highlights the differential expression signature distinguishing C9ORF72-related tissues from controls, rather than revealing unexpected clustering among individual miRNAs or target genes. These findings suggest that mutations in ALS-associated genes may disrupt miRNA biogenesis and function, potentially contributing to disease pathogenesis.

## 4 Discussion

Post-mortem tissue availability can help investigating the molecular factors implicated in the vulnerability of motor neurons in ALS, and how they influence the disease onset and clinical course.

In our study we focused on human post-mortem spinal cord tissue from two patients diagnosed with spinal onset C9ORF72-related ALS, confirmed by our G4C2 hexanucleotide repeat analysis that showed the presence of G4C2-rich repeat expansion in the tissue. At histological level, we observed degenerating motor neurons with pyknotic nuclei surrounded by coalescing vacuoles resulting in a sponge-like appearance of both gray and white matter regions, which has been previously described (Fomin et al., 2018; Scheuermann et al., 2010). Furthermore, reactive glial cells, in both white and gray spinal cord regions, suggested neuroinflammation which has been reported to occur along with motor neuron degeneration (Garofalo et al., 2020; Liao et al., 2012). By Nissl staining, we observed different size circumferential cytoplasmic and “skein-like” inclusions in motor neurons of C9ORF72-ALS patient, resembling TDP43 pathology (Shellikeri et al., 2020).

BaseScope technology revealed C9ORF72 RNA foci in the motor neuron nuclei of ventral region of spinal cord and hypoglossal and ambiguous nuclei of medulla oblongata. Interestingly, while both regions showed similar accumulation of nuclear RNA foci, overt motor neuron degeneration was predominantly observed in the spinal cord. This regional difference may reflect a greater intrinsic vulnerability of spinal motor neurons to the toxic effects of repeat RNA and/or downstream pathogenic mechanisms such as impaired RNA splicing, transport and translation impairment (Kharel et al., 2020). In contrast, neurons in the medulla oblongata may exhibit greater resilience or compensatory mechanisms that delay cell death, despite the presence of pathological RNA foci. These findings support the concept that RNA foci accumulation alone is not sufficient to trigger neurodegeneration, and that region-specific susceptibility likely plays a critical role in disease progression.

Since miRNAs play a crucial role in neurodegenerative diseases (Marcuzzo et al., 2015; Marcuzzo et al., 2014), we investigated their expression in LCM human post-mortem spinal cord tissue sections of two C9ORF72- related ALS patients and two donors deceased for cerebral hemorrhage. Based on miRNA databases, Gene Ontology enrichment analysis, and literature data we identified putative target genes potentially implicated in the pathological mechanisms underlying motor neuron degeneration. Our approach allowed the identification of two groups of miRNAs that were exclusively expressed in control and ALS tissues. Specifically, miR-30d-5p, miR-106b-5p, and miR-135a-5p were exclusively expressed in the ventral horns of healthy donors and were undetectable in the same regions of C9ORF72-related ALS patients. MiR-30d-5p was previously found to be reduced in the cerebrospinal fluid of ALS patients compared to healthy donors (Waller et al., 2017). This miRNA is involved in neuroinflammation suppression and microglia activation by targeting HOXA11 and SOCS1 genes (Chang et al., 2008; Li et al., 2022; Qin et al., 2006; Turnley et al., 2002), and of apoptosis by targeting CASP3 (Moreno-Mateos et al., 2013), a well-known gene for its role in cell death (Green and Kroemer, 2004; Schulze-Osthoff et al., 1998). One further putative target gene of miR-30d-5p is KMT2C, responsible for DNA modifications (Jozwik et al., 2016), which suggests the potential role of this miRNA in DNA damage repair. The loss of miR-30d-5p could be considered as an indirect clue of increased DNA damage in C9ORF72-related ALS.

Target gene analysis by RT-PCR identified seven putative target genes for miR-106b-5p: (i) TGF*β*R2 and SMAD4 that can induce cell death when highly expressed, by acting on the TGF-β signaling pathway (Rojas et al., 2009; Shi and Massagué, 2003); (ii) DPP9 and STAT3 that are involved in the modulation of immune mechanisms and neuroinflammation since their overexpression was associated with microglial activation (Hillmer et al., 2016; Martínez-Fábregas et al., 2018; Matheeussen et al., 2013; Röhnert et al., 2012; Sharif et al., 2021; Toral-Rios et al., 2020) and (iii) TSG101, which is implicated in vacuole and lysosome trafficking (Henne et al., 2011).

MiR-135a-5p is a stimulator of axon regrowth and plays a neuroprotective role by inhibiting apoptosis (Liu et al., 2016; van Battum et al., 2018; Wang et al., 2020). For this miRNA, five target genes were identified including FOXO1 and TXNIP that are related to apoptotic mechanisms (Andrés-Benito et al., 2017; Zhang et al., 2011), and ROCK1/2 and JAK2, which are mainly involved in the induction of neuroinflammation (Borrajo et al., 2014; Liu et al., 2023; Roser et al., 2017; Yan et al., 2012). These findings together with literature data suggest that differential expression of these miRNAs in ALS ventral horns as compared with normal tissue might be involved in the impairment of motor neurons homeostasis, survival, proliferation, and differentiation.

Our molecular analysis by RT-PCR revealed a lower expression of SOCS1 and DPP9 genes in healthy tissues. Although preliminary, these findings suggest that in healthy control tissues, the inflammatory signaling cascade leading to SOCS1 activation, as well as the metabolic pathway involving DPP9, a gene implicated in proteostasis and cellular stress responses, remains largely inactive. In contrast, in C9ORF72-related ALS tissues, both pathways appear to be upregulated, potentially reflecting an ongoing neuroinflammatory state and altered protein homeostasis associated with disease pathology. This differential activation may contribute to the molecular mechanisms underlying motor neuron vulnerability and degeneration in ALS.

Our profiling data identified two miRNAs, miR-200b-3p and miR-346, specifically expressed in C9ORF72-ALS ventral horn spinal cord tissue. MiR-200b-3p plays a crucial role in different cellular mechanisms associated with neurodegenerative processes (Fu et al., 2019; Kmetzsch et al., 2021; Pandey et al., 2015; Trümbach and Prakash, 2015). Through functional *in silico* analysis, we identified as potential target genes of this miRNA: (i) SOX2 and CREB1 that are critical for cell growth and survival (Peng et al., 2013); (ii) BAP1 and SMAD2 that are involved in ubiquitination and autophagy (Bononi et al., 2017; Eletr and Wilkinson, 2011; Scheuermann et al., 2010; Zhang et al., 2020); (iii) GRIN2A, whose altered expression is known to contribute to neurotoxicity; and (iv) TET3 that is implicated in genome modification and induction of specific gene transcription (Morris-Blanco et al., 2021).

The miR-346, which we found abnormally expressed in spinal cord of ALS patients, regulates the expression of three genes, including BCL6 that is strictly related to cell survival (Bonnefont et al., 2019; Jiang et al., 2023; Kurosu et al., 2003), TERT and GSK3β that are involved in ER stress-related responses and autophagy (Hosoi et al., 2014; Nie et al., 2016). These findings suggest that normalizing the expression of these miRNAs and their target genes, which could be potentially crucial for spinal cord motor neurons survival, might be explored as a novel therapeutic approach for C9ORF72-related ALS.

Moreover, SOX2 expression showed an increasing trend in C9ORF72-related ALS tissues compared to healthy controls, suggesting the activation of a compensatory, pro-survival response in an attempt to preserve motor neuron integrity. This increase could reflect an effort by affected neurons or surrounding glial cells to counteract the progressive loss of motor neurons characteristic of ALS.

Our study presents some limitations, including the low sample size due to the well-known difficulty of collecting well-preserved post-mortem tissues enriched in motor neurons from genetically confirmed C9ORF72 cases, as well as the absence of functional experiments to validate the role of the candidate miRNAs and corroborate the *in silico* analyses. Nevertheless, we clearly state in the manuscript the exploratory nature of our analysis and the need for future validation in larger or independent datasets. Despite these limitations, the molecular signatures we identified may represent key early alterations and provide valuable insights into the pathogenic mechanisms underlying C9ORF72-related ALS.

## 5 Conclusion

Our findings suggest that specific miRNAs and their target genes may play a role in motor neuron degeneration in C9ORF72-ALS. Importantly, restoring their expression to physiological levels may open the way to a novel therapeutic strategy aimed at slowing or even modifying disease progression.

## Data Availability

The raw data supporting the conclusions of this article will be made available by the authors, without undue reservation.

## Footnotes

1
https://www.ciidirsinaloa.com.mx/RefFinder-master/

## Glossary

ALS: Amyotrophic lateral sclerosis
sALS: Sporadic amyotrophic lateral sclerosis
fALS: Familial amyotrophic lateral sclerosis
C9ORF72: Chromosome 9 open reading frame 72
TARDBP: TAR DNA-binding protein 43
SOD1: superoxide dismutase 1
FUS: FUS RNA-binding protein
HRE: hexanucleotide repeat expansion
DPR: dipeptide repeat protein
miRNAs: microRNAs
LCM: laser capture microdissection
H&E: hematoxylin & eosin
FFPE: formalin-fixed paraffin-embedded
CASP3: caspase 3
HOXA11: Homeobox A11
SOCS1: suppressor of cytokine signaling 1
KMT2C: Lysine Methyltransferase 2C
TGFB-R2: transforming growth factor beta receptor 2
SMAD4: SMAD family member 4
TSG101: tumor susceptibility 101
STAT3: signal transducer and activator of transcription 3
DPP9: dipeptidyl peptidase 9
FOXO1: Forkhead Box O1
TXNIP: thioredoxin interacting protein
ROCK: rho associated coiled-coil containing protein kinase
JAK2: janus kinase 2
CREB1: element binding protein 1
SOX2: SRY-Box Transcription Factor 2
GRIN2A: glutamate ionotropic receptor NMDA type subunit 2A
BAP1: BRCA1 associated protein 1
TET3: tet methylcytosine dioxygenase 3
BCL6: BCL6 Transcription; Repressor
TERT: telomerase reverse transcriptase
GSK3β: glycogen synthase kinase 3 beta.
