# Supplementary material for: MicroRNA profiling in post-mortem spinal cord of C9ORF72-related ALS patients reveals molecular pathways involved in motor neuron degeneration
Source: Front Neurosci. 2026 Jan 23;20:1741065. doi: 10.3389/fnins.2026.1741065 (PMC12876226; doi:10.3389/fnins.2026.1741065)
Supplement: Supplementary file 1 [file Data_Sheet_1.DOCX]

**MicroRNA profiling in post-mortem spinal cord of C9ORF72-related ALS patients reveals molecular pathways involved in motor neuron degeneration**

Giorgia Farinazzo, Eleonora Giagnorio, Matteo Marcuzzo, Marco Cattaneo, Claudia Malacarne, Paola Cavalcante, Silvia Bonanno, Emanuela Maderna, Viviana Pensato, Cinzia Gellera, Gianluca Marucci Samanta Mazzetti, Erika Salvi, Giuseppe Lauria, Stefania Marcuzzo

**List of Supplementary Materials**

Supplementary Figures:

*Supplementary* *Figure 1.* GGGGCC hexanucleotide repeat analysis in human post-mortem tissue.

*Supplementary Figure 2*. Human post-mortem cervical spinal cord tissue sections of healthy donor subjected to laser capture microdissection (LCM).

Supplementary Table:

*Supplementary Table 1.* List of the selected target genes analyzed via PCR-RT and their respective assay IDs.

*Supplementary Table 2.* List of the exclusively expressed miRNAs in healthy donors and C9ORF72-related ALS patients with their relative PCR-RT data.

*Supplementary Table 3.* List of the selected miRNAs, target genes and biological pathways.

**1. Supplemental Figures.**

Supplementary Figure 1

*
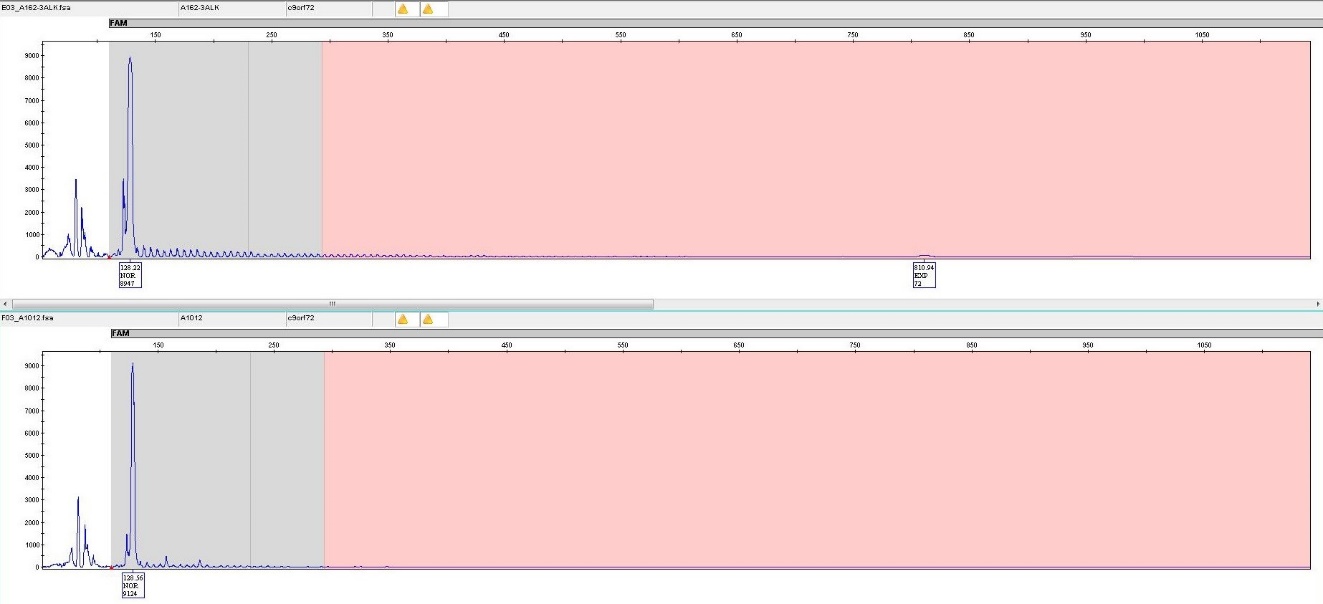
*

**Figure 1.** GGGGCC hexanucleotide repeat analysis in human post-mortem tissue. Graphs obtained from the analysis of the C9ORF72 gene using the AmplideX PCR/CE kit in ALS post-mortem tissues from two expanded patients. Graphic representation shows the saw tooth pattern.

Supplementary Figure 2


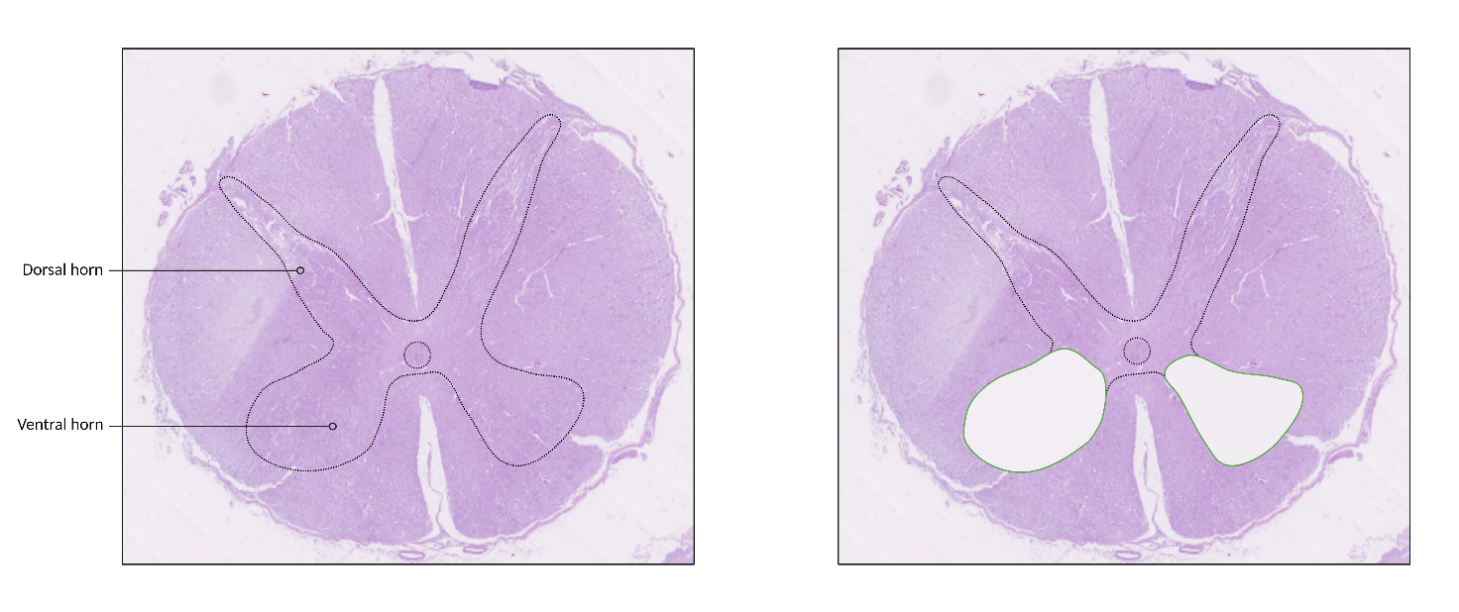


**Figure 2.** Human post-mortem cervical spinal cord tissue sections of healthy donor subjected to laser capture microdissection (LCM). Representative image showing human post-mortem cervical spinal cord tissue sections of healthy donor subjected to LCM. Grey matter is indicated by black outline and LCM area is indicated by green outline. Magnification: 10X.

**2. Supplementary Table.**

Supplementary Table 1

**Table 1.** List of the selected target genes analyzed via PCR-RT and their respective assay IDs.

| **Gene** | **Gene expression assay ID** |  | **Gene** | **Gene expression assay ID** |
| --- | --- | --- | --- | --- |
| CASP3 | Hs00234387_m1 |  | TXNIP | Hs01006897_g1 |
| KMT2C | Hs01005521_m1 |  | FOXO1 | Hs00231106_m1 |
| HOXA11 | Hs00194149_m1 |  | SOX2 | Hs04234836_s1 |
| SOCS1 | Hs00705164_s1 |  | CREB1 | Hs00231713_m1 |
| STAT3 | Hs00374280_m1 |  | GRIN2A | Hs00168219_m1 |
| TGFBR2 | Hs00234253_m1 |  | SMAD2 | Hs00998187_m1 |
| SMAD4 | Hs00929647_m1 |  | BAP1 | Hs01109276_g1 |
| TSG101 | Hs00173072_m1 |  | TET3 | Hs00896441_m1 |
| DPP9 | Hs00373589_m1 |  | BCL6 | Hs00153368_m1 |
| JAK2 | Hs01078136_m1 |  | GSK3B | Hs01047719_m1 |
| ROCK1 | Hs01127701_m1 |  | TERT | Hs00972650_m1 |
| ROCK2 | Hs00178154_m1 |  | 18S | Hs99999901_s1 |

Supplementary Table 2

**Table 2.** List of the exclusively expressed miRNAs in healthy donors and C9ORF72-related ALS patients with their relative PCR-RT data.

| **miRNA** | **Pool** | **Sample** | **Cq** | **Amp Score** | **Cq Conf** | **Amp Status** |
| --- | --- | --- | --- | --- | --- | --- |
| hsa-miR-106b-000442 | A | C9ORF-ALS 1 | 20,96 | 0,74 | 0,178 | Inconclusive |
|  |  | C9ORF-ALS 2 | 33,93 | 0,85 | 0,918 | Amp |
|  |  | HD 1 | 34,32 | 1,34 | 0,972 | Amp |
|  |  | HD 2 | 31,01 | 1,42 | 0,974 | Amp |
| hsa-miR-135a-000460 | A | C9ORF-ALS 1 | Undet. | - | 0 | No Amp |
|  |  | C9ORF-ALS 2 | Undet. | - | 0 | No Amp |
|  |  | HD 1 | 34,17 | 1,39 | 0,960 | Amp |
|  |  | HD 2 | 35,24 | 1,39 | 0,970 | Amp |
| hsa-miR-30d-000420 | B | C9ORF-ALS 1 | 35,66 | 0,74 | 0,684 | Amp |
|  |  | C9ORF-ALS 2 | Undet. | 0,34 | 0 | No Amp |
|  |  | HD 1 | 35,10 | 1,46 | 0,982 | Amp |
|  |  | HD 2 | 33,28 | 1,45 | 0,968 | Amp |
| hsa-miR-10b#-002315 | B | C9ORF-ALS 1 | Undet. | - | 0 | No Amp |
|  |  | C9ORF-ALS 2 | Undet. | - | 0 | No Amp |
|  |  | HD 1 | 35,47 | 1,17 | 0,896 | Amp |
|  |  | HD 2 | 33,79 | 1,38 | 0,981 | Amp |
| hsa-miR-127-000452 | A | C9ORF-ALS 1 | 34,26 | 0,58 | 0,481 | Amp |
|  |  | C9ORF-ALS 2 | 35,43 | 0,64 | 0,618 | Amp |
|  |  | HD 1 | 34,29 | 1,34 | 0,968 | Amp |
|  |  | HD 2 | 30,54 | 1,42 | 0,984 | Amp |
| hsa-miR-590-5p-001984 | A | C9ORF-ALS 1 | Undet. | - | 0 | No Amp |
|  |  | C9ORF-ALS 2 | Undet. | - | 0 | No Amp |
|  |  | HD 1 | 34,66 | 1,51 | 0,939 | Amp |
|  |  | HD 2 | 33,14 | 1,53 | 0,978 | Amp |
| hsa-miR-93#-002139 | B | C9ORF-ALS 1 | Undet. | - | 0 | No Amp |
|  |  | C9ORF-ALS 2 | Undet. | - | 0 | No Amp |
|  |  | HD 1 | 33,86 | 1,22 | 0,977 | Amp |
|  |  | HD 2 | 33,00 | 1,28 | 0,925 | Amp |
| hsa-miR-200b-002251 | A | C9ORF-ALS 1 | 33,79 | 1,07 | 0,891 | Amp |
|  |  | C9ORF-ALS 2 | 33,35 | 1,05 | 0,908 | Amp |
|  |  | HD 1 | Undet. | - | 0 | No Amp |
|  |  | HD 2 | 33,19 | 0,84 | 0,917 | Amp |
| hsa-miR-346-000553 | A | C9ORF-ALS 1 | 30,14 | 1,04 | 0,972 | Amp |
|  |  | C9ORF-ALS 2 | 32,11 | 1,06 | 0,938 | Amp |
|  |  | HD 1 | 31,47 | 0,98 | 0,727 | Amp |
|  |  | HD 2 | Undet. | 0,34 | 0 | No Amp |
| hsa-miR-1225-3P-002766 | B | C9ORF-ALS 1 | 28,96 | 1,09 | 0,875 | Amp |
|  |  | C9ORF-ALS 2 | 30,06 | 1,01 | 0,949 | Amp |
|  |  | HD 1 | 26,83 | 0,92 | 0,473 | Amp |
|  |  | HD 2 | 30,54 | 0,87 | 0,798 | Amp |

Supplementary Table 3

**Table 3.** List of the selected miRNAs, target genes and their biological pathways.
